# The Complex Determinants of Older Adult Behavioral Health: A State Level Overview and Response

**DOI:** 10.1093/geroni/igaf122.1978

**Published:** 2025-12-31

**Authors:** Walter Dawson, Paula Carder, Allyson Stodola, Lindsey Smith, Karen Cellarius, Leah Brandis

**Affiliations:** Oregon Health & Science University, Portland, Oregon, United States; Portland State University, Portland, Oregon, United States; Portland State University, Portland, Oregon, United States; Oregon Health & Science University, Portland, Oregon, United States; Portland State University, Portland, Oregon, United States; Oregon Health & Science University, Portland, Oregon, United States

## Abstract

To address the multiple, complex determinants of older adult behavioral health, Oregon established the first state-level center of excellence in the U.S. with a specific focus on this population through a state-university-community partnership. Previous research has shown that comprehensive care for older adults with behavioral health needs is impeded by the workforce shortage of generalist BH clinicians and a severe workforce shortage of clinicians trained to work with older adults. In the years ahead it is projected there simply will not be enough behavioral health providers who specialize in gero focused-fields to meet the needs of an increasingly aging population. This lack of trained professionals, in turn, leads to a lack of awareness, screening, and proper assessment of the unique behavioral health needs of older adults. Building on Oregon’s long history of innovation with the services and care of older adults, the Oregon Center of Excellence for Behavioral Health and Aging (OCEBHA) was conceptualized by the state health authority and initiated using block grant funds from the Substance Abuse and Mental Health Services Administration (SAMHSA). OCEBHA seeks to expand the capacity of health and social programs and providers to deliver behavioral health services for older adults with serious mental illness and substance use disorders through translational research, workforce development, and policy innovation. The focused approach taken by Oregon can serve as a catalyst for further innovation in behavioral health and aging service delivery.

